# Relationships between stem cell exhaustion, tumour suppression and ageing

**DOI:** 10.1038/sj.bjc.6604029

**Published:** 2007-10-09

**Authors:** Y Ruzankina, E J Brown

**Affiliations:** 1 Department of Cancer Biology, Abramson Family Cancer Research Institute, University of Pennsylvania School of Medicine, 421 Curie Blvd., Philadelphia, PA 19104-6160, USA

**Keywords:** ageing, stem cells, DNA damage, oncogenes

## Abstract

Ageing is a complex process characterised by a variety of disorders associated with general organismal decline and an inability to maintain tissue homoeostasis. As described in this review, recent studies indicate that ageing may be caused, in part, by the depletion of stem and progenitor cells that govern tissue renewal. The potential causes of stem and progenitor cell attrition are numerous; however, a commonly accepted theory is that these cells are lost as a result of naturally occurring DNA damage and the obligate checkpoint responses that follow. Failure to launch appropriate responses to DNA damage is strongly associated with cancer initiation and progression. Therefore, it is at this nexus, the response to DNA damage, that an important organismal fate may be determined: to degrade regenerative potential for the purpose of preventing cancer. According to this viewpoint, ageing may be the unfortunate mark of successful cancer suppression in stem cells and other cell types. In this review, we will describe how degeneration of tissue renewal capacity links ageing and cancer suppression.

Cancer and ageing are well correlated. That is, cancer risk increases in a manner that is, generally speaking, coincident with increasing age (Figure 1). Furthermore, both cancer and ageing have been proposed to be attributable to a similar fundamental cause: the accumulation of DNA damage (Hasty *et al*, 2003; Lombard *et al*, 2005). Although cancer and age-related diseases are well correlated, there is an exception; a statistically significant decrease in cancer is observed in human populations beyond 85 years of age (Figure 1). This apparent decrease could be the consequence of selection for those individuals that are devoid of cancer-predisposing genes. However, an increasing body of evidence supports a different hypothesis: that processes linked to ageing, both cellular and organismal, may suppress cancer. This theory taps into several converging lines of evidence. The first line demonstrates that stem and progenitor cells rely on the induction of tumour-suppressing genes, such as p16^INK4A^ and p53, to suppress cell growth in the event of DNA damage or hyperactive growth factor signalling. By inducing permanent cell cycle arrest (senescence) or programmed cell death (apoptosis), these genes suppress tumourigenesis, but may also consequently degrade stem and progenitor cell pools. Therefore, over the long term, the response to DNA damage and replicative stress may lead to exhaustion of tissue renewal capacity. Another line of evidence indicates that stem and progenitor cells exhaustion can contribute substantially to the process of ageing, as determined by observations of stem and progenitor cells attrition in mouse models in which genome maintenance is compromised. Together, these studies indicate that hyperactive growth factor signalling and accumulated DNA damage are the initial causes of a branching chain of events that leads to ageing, when tumour-suppressive mechanisms are effective, and cancer, when such mechanisms fail.

## STEM CELL EXHAUSTION AND AGEING

Ageing is a complex process generally characterised by reduced cellular function and an inability to maintain tissue homoeostasis. Age-related changes include slowed wound-healing responses and haematopoietic recovery rates, osteoporosis, kyphosis, hair loss and graying, reduced fertility, muscle loss, neurodegeneration and cancer (Hasty *et al*, 2003; Kipling *et al*, 2004; Lombard *et al*, 2005). These phenotypes are not uniformly expressed in all individuals and are not strictly age associated in all cases (eg alopecia); however, the appearance of these phenotypes in general is strongly correlated with advancing age. A number of human progeroid syndromes (Werner, Cockayne, Hutchinson-Gilford, trichothyodystrophy, dyskeratosis congenita, ataxia-telangiectasia) and mutant mouse models (below) recapitulate some aspects of normal ageing and in many instances produce extreme examples of these disorders (Hasty *et al*, 2003; Kipling *et al*, 2004; Lombard *et al*, 2005). Predominantly, the genes mutated in these examples are required for genome maintenance, either in the form of DNA repair or in the cell cycle-regulatory response to DNA damage. Therefore, increased genomic instability is sufficient to speed the onset of age-related disorders. However, while DNA damage may be a well-accepted root cause of ageing, how DNA damage causes ageing at the organismal level is still unclear. As noted above, decreased tissue maintenance and rates of regeneration provide a unifying thread between many age-related phenotypes. Although DNA damage affects many cell types, including terminally differentiated cells, the depletion of stem and progenitor cell pools would be expected to cause a potent effect on regeneration of many tissues (Van Zant and Liang, 2003; Rando, 2006). Recent evidence using targeted deletions of important genome-maintenance regulators in mice lends credence to the hypothesis that stem and progenitor cell exhaustion leads to age-related phenotypes.

The age-associated disorders described above have been observed in a number of mouse models, although to varying degrees. These models include Ku86^−/−^, XPD^R722W/R722W^, BRCA1^Δ11/Δ11^p53^+/−^, XRCC4^−/−^p53^S18A/S23A^, LIG4^Y288C^, Rad50^S/S^, Terc^−/−^, Wrn^−/−^Terc^−/−^ and those expressing putatively hypermorphic variants of p53 (Hasty *et al*, 2003; Lombard *et al*, 2005; Chao *et al*, 2006). Tissue regenerative failures are observed in some form or another in each of these models, and recent reports indicate that mutation of many of these same genome-maintenance regulators leads to decreased stem cell number and potential with age (Ito *et al*, 2004; Nijnik *et al*, 2007; Rossi *et al*, 2007). In addition, recent study of haematopoietic stem cells from aged normal mice revealed a decline in stem cell regenerative potential and epigenetic downregulation of genes involved in the maintenance of genomic integrity (Chambers *et al*, 2007). Although mutant mouse models may not fully reflect all of the events happening during normal mammalian ageing, the observed correlations may help us understand the mechanisms by which natural ageing can occur.

The specific cause of regenerative failures has been predominantly studied in mice in which Terc, a telomerase co-factor, has been mutated. After three generations in the absence of telomerase (G3), Terc^−/−^ mice are born with significantly shortened telomeres, which causes chromosome end fusions and persistent genomic instability through the bridge-breakage-fusion cycle (Rudolph *et al*, 1999). At G3 and beyond Terc^−/−^ mice exhibit impaired haematopoiesis, atrophy of the intestinal epithelium, decreased spermatogenesis and reduced regenerative capacity in response to wounds and haematopoietic ablation (Rudolph *et al*, 1999). Telomerase loss in combination with deletion of genes that regulate alternative telomere lengthening, such as the gene mutated in Werner and Bloom syndromes, accelerates telomere shortening. These compound mutants have accentuated degenerative phenotypes, which appear at earlier generation times, and suffer additional pathologies, such as bone loss and kyphosis (Chang *et al*, 2004; Du *et al*, 2004). Importantly, stem cells, which normally express telomerase, are particularly sensitive to telomerase loss (Flores *et al*, 2006; Hiyama and Hiyama, 2007). Thus, increased genomic instability from telomerase absence in haematopoietic stem cells (HSCs), spermatogonia and progenitor cells in the intestinal crypts is likely to be the cause of homeostatic failure in the bone marrow, testes and intestines, respectively (Rudolph *et al*, 1999; Choudhury *et al*, 2007). Together, these data are consistent with the notion that decreased genome maintenance in stem and progenitor cells accelerates tissue degeneration with age.

Recent studies imply that stem cell attrition in Terc^−/−^ mice is not so much the product of DNA damage itself, but rather of the response to DNA damage. DNA damage leads to the activation of checkpoint signalling pathways mediated by the ATM and Rad3 related (ATR) and Ataxia-telangiectasia mutated (ATM) protein kinases, which, among other things, phosphorylate and stabilise the p53 transcription factor (Kastan and Bartek, 2004). Activated p53 consequentially stimulates the expression of cell cycle regulators, such as p21, and, in some cell types, initiates cell death programs via upregulation of pro-apoptotic genes. Importantly, elimination of p21 largely rescues haematopoiesis and intestinal villi generation in late generation Terc^−/−^ mice (Choudhury *et al*, 2007). Moreover, p21 absence returns HSCs (CD34^lo/−^Lin^−^Sca1^+^c-Kit^hi^) to normal numbers in G4 Terc^−/−^ mice and dramatically enhances HSC potential for generating downstream progenitors. The number and proliferative potential of intestinal progenitors in Terc^−/−^ mice were also dramatically enhanced by elimination of p21. Interestingly, these degenerative phenotypes were lessened by p21 absence without rescuing either telomere attrition or DNA damage, as measured by increased H2AX phosphorylation (Choudhury *et al*, 2007). These data strongly argue that the response to DNA damage, not DNA damage *per se*, can cause a decrease in tissue renewal capacity.

It is important to point out that stem cells normally express telomerase; therefore, they may not suffer telomere attrition during normal ageing, in contrast to other cells. Besides telomere loss, DNA damage occurs constitutively in all cell types as a by-product of normal metabolic respiration and from unavoidable failures during DNA replication. Thus, one model of ageing predicts that replicative and oxidative stress in the course of stem cell proliferation leads to slow stem cell attrition throughout adulthood, and this attrition ultimately degrades tissue homoeostasis. One way to test such a model would be to ablate the proliferative potential of a large fraction of cells in young adult animal while leaving the remaining fraction of cells relatively untouched. Taking a large fraction of cells out of the proliferative pool early in life at one stroke should shorten the length of time in which a particular stem cell compartment will be able to continue to contribute to tissue renewal and, thus, would accelerate ageing. Such a study has recently been performed by deleting an essential genome-maintenance and checkpoint regulator, ATR, in adult mice (Ruzankina *et al*, 2007).

The ATR protein kinase senses problems during DNA replication and, in the event of stalled DNA replication, maintains replication fork stability and prevents cell cycle progression. Stalled DNA replication appears to be a common event during normal cellular proliferation, since elimination of ATR in cultured proliferating cells results in catastrophic loss of genome integrity, cell cycle exit and apoptosis (Brown and Baltimore, 2000; Cortez *et al*, 2001; Casper *et al*, 2002; Brown and Baltimore, 2003). While ATR is essential for embryonic development (Brown and Baltimore, 2000), mosaic deletion of ATR in adult mice (ATR^mKO^) via a tamoxifen-inducible form of Cre recombinase leads to the rapid appearance of a number of age-related phenotypes including hair graying, alopecia, kyphosis, osteoporosis, premature thymic involution, loss of spermatogenesis and increased tissue fibrosis (Ruzankina *et al*, 2007). Consistent with the importance of stem cells in preventing age-related disease, thymic involution, alopecia and hair graying in ATR^mKO^ mice were associated with dramatic reductions in tissue-specific stem and progenitor cells and exhaustion of tissue renewal and homeostatic capacity.

Importantly, the phenotypes observed in ATR^mKO^ mice do not appear to be caused by the persistence of ATR mutant cells. Following Cre recombination, ATR knockout cells were rapidly depleted from affected tissues. This depletion was attributable to the proliferative failure of ATR knockout cells, which is most likely due to loss of genome maintenance during DNA replication (Brown and Baltimore, 2003; Ruzankina *et al*, 2007). Concomitant with the depletion of ATR-deleted cells, most of these same tissues were rapidly reconstituted with the small percentage of ATR-expressing cells that escaped lox recombination. Therefore, the long-term failure of stem and progenitor cells in ATR^mKO^ mice took place despite ample reconstitution with ATR-expressing cells. These results suggest that elimination of ATR knockout cells leads to the proliferative exhaustion of the remaining ATR-expressing cells, which in turn causes degeneration of tissue renewal capacity (Krishnamurthy and Sharpless, 2007; Ruzankina *et al*, 2007).

Many questions remain to be answered using these mouse models and others. For examples, it is possible that ATR deletion also affects the niches that maintain stem cells; thus, ATR-expressing stem cell pools may be depleted by both cell intrinsic and extrinsic mechanisms. Intrinsic HSC defects and niche degeneration have each been observed in telomerase-deficient mice (Choudhury *et al*, 2007; Ju *et al*, 2007). In addition, while telomerase deficiency causes a relatively slow, but persistent, accumulation of DNA damage, ATR elimination leads to an immediate catastrophic loss of genome integrity and clearance of the damaged cells (Ruzankina *et al*, 2007). At present, it is not clear to what extent the DNA damage response plays a role in the onset of age-related phenotypes in ATR^mKO^ mice and if p53 and p21 deficiency may rescue the appearance of these phenotypes.

These data indicate that the loss of stem cell function as a result of DNA damage correlates with and may be sufficient to cause at least some aspects of normal ageing. In particular, it appears that the response to DNA damage, not DNA damage *per se*, may cause the loss of regenerative capacity. At first appearance, the advantage of preventing tissue renewal on an organismal level is not clear, until another effect of DNA damage is considered.

## TUMOUR-SUPPRESSING MECHANSIMS DEGRADE TISSUE RENEWAL CAPACITY

As described above, stem cells and their downstream progeny are subjected to DNA damage from a broad range of sources including the by-products of normal metabolic respiration and unavoidable failures during DNA replication. In addition, hyperactive proliferative signalling via proto-oncogene expression and activation (eg Bmi1, Wnt, *β*-catenin, Shh) are also a normal part of cell renewal and have been shown to be keenly important for stem cell maintenance (Pardal *et al*, 2005). Both DNA damage and uncontrolled proliferative signalling are key elements to cancer initiation and progression. Therefore, because DNA damage and hyperactive proliferative signalling are unavoidable consequences of living, the senescence and apoptotic programs initiated by these stimuli are essential barriers to tumourigenesis in multicellular organisms.

Two of the best characterised regulators of senescence and apoptosis in response to DNA damage and proto-oncogneic signalling are p16^INK4A^ and p53. Each is induced by DNA damage and oncogenic stress and has been shown to suppress cancer in mouse models. Disruption of p16^INK4A^ in mice leads to increased risk of osteosarcomas, histiocytic lymphomas and melanomas and inactivation of INK4A in humans is found in approximately 30% of the most common cancers (Kim and Sharpless, 2006). A similar high correlation between p53 loss and increased cancer incidence has been observed in humans and p53 mutant mice (Kastan and Bartek, 2004). The importance p16^INK4A^ and p53 in tumour suppression in the absence of abnormal environmental factors indicates that these genes are utilised frequently throughout life and that cancer incidence is indicative of their failure.

Because activation of p16^INK4A^ and p53 affects continued cellular proliferation through either senescence or apoptosis, the constant activation of p16^INK4A^ and p53 could influence long-term tissue homoeostasis. Accumulation of senescent cells has long been viewed as potential cause of ageing (Campisi, 2005), and it has been shown that abnormally high levels of apoptosis achieved by genetically altering mitochondrial function leads to the premature appearance of age-related phenotypes (Kujoth *et al*, 2005). As described below, recent evidence supports the hypothesis that effective cancer suppression ultimately degrades tissue renewal capacity. According to this model, one would expect that hyperactivation of tumour-suppressing pathways in the absence of increased DNA damage or proto-oncogenic signalling would be sufficient to cause aspects of ageing. In contrast, loss of tumour-suppressing pathways may slow tissue degeneration. Each of these expectations has been at least partly fulfilled by the generation and analysis of mouse models that alter the activity and/or expression of p53 and p16^INK4A^.

In one mouse model, a serendipitous deletion in p53 (p53^m^) was generated in the course of conventional gene targeting, leading to a putative 24 kDa N-terminally truncated protein (Tyner *et al*, 2002). In the background of a p53 null allele (p53^m/−^), p53^m^ appears to be non-functional; however, when in the presence of a wild-type copy of p53 (p53^+/m^), the p53^m^ allele causes a small but detectable hypermorphic effect on the transcription of p53 target genes and suppression of oncogene-mediated transformation in culture. Presumably, the effect of the 24 kDa protein is mediated through higher order complex formation with wild-type p53 in p53^+/m^ mice. p53 hyperactivation in these mice is accompanied by a ∼20% shorter median lifespan and the premature appearance of several age-related phenotypes, including osteoporosis, kyphosis, generalised organ atrophy, impaired wound healing, hair re-growth defects and slowed recovery following myeloablation. While it has been shown that the deletion leading to this truncation also causes haploinsufficient loss of 23 nearby genes (Gentry and Venkatachalam, 2005), a careful analysis of the phenotypes observed in p53^+/m^, p53^m/−^ p53^+/−^ and p53^−/−^ mice suggests that the premature ageing phenotypes in p53^+/m^ mice are most likely related to the p53 truncation (Vijg and Hasty, 2005). In addition, phenotypes consistent with those observed in p53^+/m^ mice have been obtained by expressing a naturally occurring N-terminally truncated isoform of p53 called p44 (Maier *et al*, 2004). Transgenic p44 mice manifest several aspects of premature ageing as well as a significantly reduced lifespan (32 week median). Consistent with the hypothesis that p53 activity suppresses cancer initiation and progression, p53^+/m^ mice exhibit decreased cancer incidence (6%) in comparison to wild-type control mice (45%), although this incidence may be underrepresented due to decreased longevity (Tyner *et al*, 2002). Importantly, a recent report by the same group has gone on to show that the p53^+/m^ mice have reduced HSC number and proliferative potential and that p53 haploinsufficiency (p53^+/−^) appears to lead to increased HSC number and potential in late age mice (Dumble *et al*, 2007). These studies indicate that aberrant hyperactivation of the p53 pathway can cause the premature appearance of age-related phenotypes.

It is important to note that, in contrast to aberrant p53 hyperactivation, an additional copy of p53 under normal regulatory control (super-p53 mice) and reduced expression of the p53 inhibitor Mdm2 do not accelerate ageing (Garcia-Cao *et al*, 2002; Mendrysa *et al*, 2006). Furthermore, the super-p53 transgene in combination with an extra copy of the Arf locus (super-p53/super-Arf) protects mice against oxidative DNA damage and cancer and extends lifespan (Matheu *et al*, 2007). It is conceivable that increased quantities of p53 under normal regulatory control can enhance the clearance and suppress the accumulation of mutant cells without causing the wholesale loss of all cell types, such as has been proposed to occur from aberrant regulation of p53 (p53^+/m^). Together, these studies indicate that the cause and level of p53 activation strongly influence the development of age-related phenotypes.

Like p53, INK4A is upregulated in response to oncogenic stress and DNA damage, although with kinetics that differ from p53. The immediate response to DNA damage and oncogenic stress involves the p53-p21 transcriptional axis; however, once these factors wane, p16^INK4A^ levels begin to rise to cause irretrievable cell cycle exit through senescence (Campisi, 2005; Kim and Sharpless, 2006). In accord with 16^INK4A^-mediated senescence playing an important role in ageing, 16^INK4A^ has been shown to be upregulated in various tissues in aged humans and rodents, and overexpression of p16^INK4A^ in mice to a degree observed with ageing leads to decreased *β*-islet regeneration (Krishnamurthy *et al*, 2004; Krishnamurthy *et al*, 2006; Ressler *et al*, 2006). If p16^INK4A^ upregulation is a cause of age-associated loss of regenerative capacity, then one would expect that deletion of p16^INK4A^ would suppress stem and progenitor cell attrition and tissue degeneration with age. For some tissues, this expectation has been fulfilled, at least in part. Age-related decline in proliferation of the forebrain progenitors, pancreatic *β*-islet cells and HSCs were rescued significantly upon deletion of p16^INK4A^ in mice, as were regeneration of the corresponding tissues (Janzen *et al*, 2006; Krishnamurthy *et al*, 2006; Molofsky *et al*, 2006). These studies strongly indicate that naturally occurring upregulation of p16^INK4A^ levels with age form a rate-limiting block in tissue regeneration and that deletion of p16^INK4A^ alone is sufficient to remove at least part of this blockade. Importantly, age-related attrition was not rescued in all stem and progenitor cell types in which p16^INK4A^ was deleted, indicating that reduced proliferative potential of some cell types with age does not rely on p16^INK4A^ (Molofsky *et al*, 2006) and that other rate-limiting factors may be involved. In aggregate, these studies imply that the activation of senescent and apoptotic signalling pathways, putatively resulting from unavoidable DNA damage and proto-oncogenic stress, can lead to reduced tissue regenerative capacity with age.

## DOES AGEING SUPPRESS CANCER?

**Figure 2:**
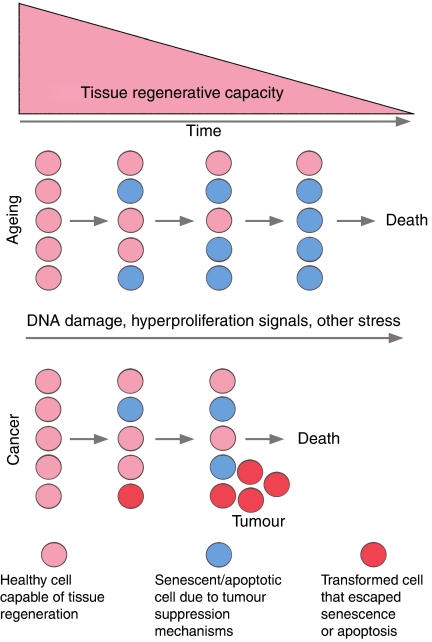
Relationship between cancer and ageing. DNA damage and proto-oncogenic stress lead to activation of senescence or apoptotic programs, which may eventually deplete adult stem cell pools and degrade tissue renewal capacity. Cells that escape senescence and apoptosis are permitted to continue cell division and undergo transformation, leading to the development of cancer.

While cancer and ageing are generally correlated, it may be that this relationship is simply rooted in common events, for example, DNA damage and proto-oncogenic signalling. According to the viewpoint described herein, it may be the effectiveness of the response to these events that determines the diametrically opposite outcomes of decreased tissue regeneration and tumourigenesis (Figure 2). If tumour-suppressing mechanisms, such as apoptosis and senescence, have been overly effective throughout life, then the consequential decrease in regeneration rates might also decrease the total output of newly generated cells that can progress to cancer. Thus, the small but statistically significant decline in cancer incidence in late ageing individuals may be the product of decreased regenerative rates and stem cell numbers. While reports in support of this hypothesis are scattered, two examples discussed herein are p53^+/m^ and ATR^mKO^ mice, which have reduced regenerative potential and appear to have a decreased tumour risk in comparison to controls (Tyner *et al*, 2002; Ruzankina *et al*, 2007). It is also possible that loss of stem and progenitor cells themselves may decrease the pool of cells available for transformation, according to the hypothesis that some cancers are derived directly from these cell types (Ward and Dirks, 2007). For example, mice disrupted for CD34, a marker of hair follicle bulge stem cells, display a significant delay in follicle cycling and, in contrast to wild-type mice, do not develop skin papillomas in response to 7,12-dimethylbenz(a)anthracene (DMBA) and 12-O-tetradecanoylphorbol-13-acetate (TPA), although DNA adduct formation was similar in both strains (Trempus *et al*, 2007). While the above studies suggest that decreased regeneration rates affect cancer incidence, a great deal remains to be done to substantiate this hypothesis.

## CONCLUSION

Recent studies suggest that aspects of normal ageing may be caused by decreased stem cell regenerative potential. This decrease in regenerative potential may be the consequence of the unavoidable accumulation of DNA damage and hyperactive growth factor signalling, which in turn initiate tumour-suppressive mechanisms designed to prevent uncontrolled cell proliferation. These mechanisms include apoptotic and senescence programs regulated by tumour suppressor genes, such as p16^INK4A^, p53 and others. Thus, anti-cancer mechanisms may contribute to decline in stem cell function and may inadvertently promote affects that we know as ageing. If cancer suppression and ageing are intimately linked, the question arises whether it will be possible to promote tumour-suppressive responses without leading to accelerated ageing. The recent study by Matheu *et al* (2007) suggests that such treatments may be possible so long as they selectively enhance responses to DNA damage, rather than non-selectively inducing p53 in all cells and causing inappropriate stem cell attrition. Other interventions to combat cancer and ageing may include the prevention of DNA damage (eg antioxidants), stem cell transplantation and regulation of the self-renewal of existing stem cells. Of course, for such interventions to be practical, additional studies will be necessary to further confirm and explore the connections described herein between stem cell exhaustion, tumour suppression and ageing.

## Figures and Tables

**Figure 1:**
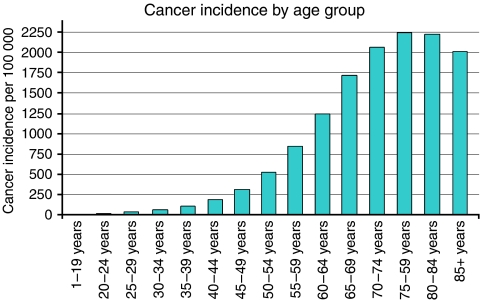
Cancer incidence increases with age; however, there is a significant decrease in cancer incidence after the age of 85. Cancer incidence by age group based on Cancer Registry Public Information Data: 1999–2002, WONDER Online Database, United States Department of Health and Human Services (http://wonder.cdc.gov/).
